# Olmutinib (BI1482694/HM61713), a Novel Epidermal Growth Factor Receptor Tyrosine Kinase Inhibitor, Reverses ABCG2-Mediated Multidrug Resistance in Cancer Cells

**DOI:** 10.3389/fphar.2018.01097

**Published:** 2018-10-09

**Authors:** Wei Zhang, Ying-Fang Fan, Chao-Yun Cai, Jing-Quan Wang, Qiu-Xu Teng, Zi-Ning Lei, Leli Zeng, Pranav Gupta, Zhe-Sheng Chen

**Affiliations:** ^1^Institute of Plastic Surgery, Weifang Medical University, Weifang, China; ^2^Department of Pharmaceutical Sciences, College of Pharmacy and Health Sciences, St. John’s University, Queens, NY, United States; ^3^Department of Hepatobiliary Surgery, Zhujiang Hospital, Southern Medical University, Guangzhou, China; ^4^MOE Key Laboratory of Bioinorganic and Synthetic Chemistry, School of Chemistry, Sun Yat-sen University, Guangzhou, China

**Keywords:** multidrug resistance (MDR), ATP-binding cassette (ABC) transporter, tyrosine kinase inhibitor (TKI), olmutinib, breast cancer resistance protein (BCRP/ABCG2)

## Abstract

The main characteristic of tumor cell resistance is multidrug resistance (MDR). MDR is the principle cause of the decline in clinical efficacy of chemotherapeutic drugs. There are several mechanisms that could cause MDR. Among these, one of the most important mechanisms underlying MDR is the overexpression of adenosine triphosphate (ATP)-binding cassette (ABC) super-family of transporters, which effectively pump out cytotoxic agents and targeted anticancer drugs across the cell membrane. In recent years, studies found that ABC transporters and tyrosine kinase inhibitors (TKIs) interact with each other. TKIs may behave as substrates or inhibitors depending on the expression of specific pumps, drug concentration, their affinity for the transporters and types of co-administered agents. Therefore, we performed *in vitro* experiments to observe whether olmutinib could reverse MDR in cancer cells overexpressing ABCB1, ABCG2, or ABCC1 transporters. The results showed that olmutinib at 3 μM significantly reversed drug resistance mediated by ABCG2, but not by ABCB1 and ABCC1, by antagonizing the drug efflux function in ABCG2-overexpressing cells. In addition, olmutinib at reversal concentration affected neither the protein expression level nor the localization of ABCG2. The results observed from the accumulation/efflux study of olmutinib showed that olmutinib reversed ABCG2-mediated MDR with an increasing intracellular drug accumulation due to inhibited drug efflux. We also had consistent results with the ATPase assay that olmutinib stimulated ATPase activity of ABCG2 up to 3.5-fold. Additionally, the molecular interaction between olmutinib and ABCG2 was identified by docking simulation. Olmutinib not only interacts directly with ABCG2 but also works as a competitive inhibitor of the transport protein. In conclusion, olmutinib could reverse ABCG2-mediated MDR. The reversal effect of olmutinib on ABCG2-mediated MDR cells is not due to ABCG2 expression or intracellular localization, but rather related to its interaction with ABCG2 protein resulting in drug efflux inhibition and ATPase stimulation.

## Introduction

The main characteristic of tumor cell resistance is MDR, in which cancer cells exhibit a cross-resistant phenotype against multiple unrelated drugs that are structurally and/or functionally different and may also have varying molecular targets (Saraswathy and Gong, 2013). MDR is the main cause of the decline in clinical efficacy of chemotherapeutic drugs. There are several mechanisms that could cause MDR, such as reduced uptake of drugs, overexpression of energy-dependent efflux proteins, increased efflux of drugs by drug transporters, inhibition of apoptosis, activation of DNA repair mechanisms, cell cycle arrest and modification of cell cycle checkpoints (Gillet and Gottesman, 2010; Hu et al., 2016; Kartal-Yandim et al., 2016; Pan et al., 2016). Among these, one of the most important mechanisms underlying MDR is the overexpression of the adenosine triphosphate (ATP)-binding cassette (ABC) super-family of transporters, which effectively pump out cytotoxic agents and targeted anticancer drugs across the cell membrane (Zhang et al., 2017; Fan et al., 2018). ABC transporters that cause drug resistance are currently divided into three main categories: ABCB, ABCC, and ABCG. Among them, ABCB1 (P-glycoprotein, P-gp), ABCC1 (MDR protein 1, MRP1), and ABCG2 (breast cancer resistance protein, BCRP) are the most common ones (Strouse et al., 2013; Miklos et al., 2015).

P-gp was the first ABC transporter that was isolated from colchicine-resistant Chinese hamster ovary cells by Juliano and Ling (1976). P-gp pumps substrates out of tumor cells through an ATP-dependent mechanism (Juliano and Ling, 1976; Kartner et al., 1983; Krishna and Mayer, 2000). MRP1 was first reported in 1992 as the mediator of acquired drug resistance in a small cell lung cancer cell line selected by repeated exposure to doxorubicin (Cole et al., 1992; Fletcher et al., 2016). Although associated with drug resistance properties, it also has been identified as an organic anion transporter in its normal physiological role (Borst et al., 1997). BCRP, encoded by ABCG2, was the third member of the ABC transporter family that was identified (Doyle et al., 1998; Miyake et al., 1999; Robey et al., 2018). A number of chemotherapeutic agents, such as MX, 9-aminocamptothecin, topotecan, irinotecan and SN-38 have been shown to be transported by BCRP (Robey et al., 2007). ABC transporters mainly contribute to MDR by altering drug absorption, distribution, excretion and metabolism (Kathawala et al., 2017). Numerous studies have found that ABC transporters are over expressed in many tumor tissues. For this reason, reversal of MDR caused by ABC transporters is one of the main strategies for tumor treatment. Researchers have been working to find new ways to inhibit ABC transporters and re-sensitize cancer cells to chemotherapeutic drugs (Kathawala et al., 2017).

Tyrosine kinases (TKs) are widely expressed in cells, and they play important roles in various cellular processes. The expression of TKs is related to the proliferation, differentiation, migration, apoptosis, angiogenesis, and metastasis of cancer cells in key signaling events/pathways (van der Geer et al., 1994; Pytel et al., 2009; Shukla et al., 2012). The phosphatidyl inositol-3-kinase (PI3K)/AKT, protein kinase C (PKC) family, and mitogen-activated protein kinase (MAPK)/Ras signaling cascades are activated by TKs and play important roles in cell proliferation and homeostasis (Faivre et al., 2006). TKs can catalyze the transfer of a phosphate group from ATP to target proteins. Therefore, TKIs have become one of the effective targets in cancer treatments (Dohse et al., 2010; Russo et al., 2017). TKIs have achieved significant clinical efficacy as anti-tumor agents. Among them, small-molecule TK inhibitors are the most promising new drugs and have shown good prospects in both clinical and experimental treatments (Chen et al., 2017; Hocker et al., 2017; Chikhale et al., 2018; Parikh and Ghate, 2018).

In recent years, studies have found that ABC transporters and several TKIs interact with each other (e.g., imatinib, sunitinib, nilotinib, and gefitinib). On the one hand, ABC transporters can cause a decrease in the function of TKIs, leading to the occurrence of MDR (Beretta et al., 2017). Parts of TKIs can be the substrates of ABC transporters. The ABC transporters pump them out of the cell and cause drug resistance (Herbrink et al., 2015). On the other hand, more and more studies have shown that some TKIs can reverse ABC transporter–mediated MDR (Ma et al., 2014). The reversal mechanisms include inhibiting the protein expression of ABC transporters, inhibiting the protein pumping function and other signaling pathways (Zhang et al., 2016a,b; Beretta et al., 2017; Kathawala et al., 2017).

**FIGURE 1:**
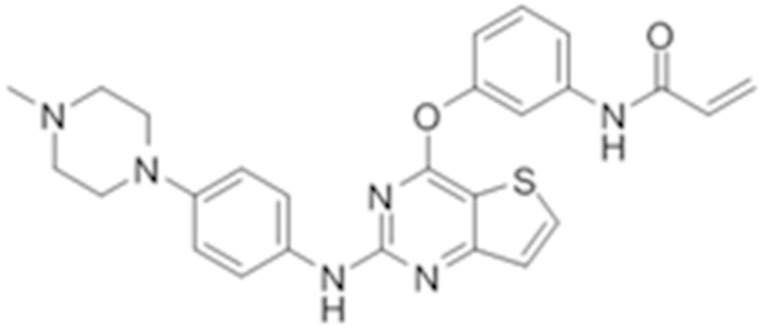
The chemical structure of olmutinib.

Olmutinib (HM61713/BI1482694) is a novel third-generation that is orally active and selectively inhibits EGFR mutations (**Figure 1**), including both activating mutations and T790M, but not EGFR wild-type (Kim, 2016). It was approved in South Korea on May 13, 2016 for the treatment of patients with locally advanced or metastatic EGFR T790M mutation-positive NSCLC previously treated with an EGFR TKI (Singh and Jadhav, 2018). At present, olmutinib is waiting for the phase III clinical efficacy observation.

Tyrosine kinase inhibitors may behave as substrates or inhibitors of ABC transporters depending on the expression of specific pumps, drug concentration, affinity for transporters and types of co-administered agents (Beretta et al., 2017). Therefore, we performed *in vitro* experiments to evaluate if olmutinib could reverse MDR in cancer cells overexpressing ABCB1, ABCG2, or ABCC1 transporters.

## Materials and Methods

### Chemicals and Reagents

Dulbecco’s modified Eagle’s medium (DMEM), fetal bovine serum (FBS), bovine calf serum (BS), penicillin/streptomycin and trypsin 0.25% were purchased from Hyclone (GE Healthcare Life Sciences, Pittsburgh, PA, United States). 10X solution of phosphate buffered saline (PBS), SN-38, and Alexa Fluor 488 conjugated rabbit anti-mouse IgG secondary antibody were purchased from Thermo Fisher Scientific Inc. (Rockford, IL, United States). The monoclonal antibodies for ABCG2 (BXP-34), paclitaxel, vincristine, vinblastine, cisplatin, MX, verapamil, 3-(4, 5-dimethylthiazol-yl)-2, 5-diphenyltetrazolium bromide (MTT), dimethyl sulfoxide (DMSO), propidium iodide (PI), and Triton X-100, were obtained from Sigma Chemical Co. (St. Louis, MO, United States). Ko-143 and MK-571 were products from Enzo Life Sciences (Farmingdale, NY, United States). Monoclonal antibodies sc-47778 (against β-actin) and secondary HRP-labeled rabbit anti-mouse IgG were purchased from Santa Cruz Biotechnology, Inc. (Dallas, TX, United States). [^3^H]- MX) (2.5 Ci/mmol) was purchased from Moravek Biochemicals (Brea, CA, United States). Liquid scintillation cocktail was a product from MP Biomedicals, Inc. (Santa Ana, CA, United States). All other chemicals were purchased from Sigma Chemical Co. (St. Louis, MO, United States).

### Cell Lines and Cell Culture

The non-small cell lung cancer cell line NCI-H460 and its drug-resistant ABCG2-overexpressing NCI-H460/MX20 cells, which was maintained in medium with an addition of 20 nM MX, were kindly provided by Drs. Susan Bates and Robert Robey (NIH, Bethesda, MD, United States). The human epidermal carcinoma cell line KB-3-1 and its drug-resistant ABCB1-overexpressing KB-C2 cells, which were cloned from KB-3-1 and maintained in medium with 2 mg/ml of colchicine, and its drug-resistant ABCC1-overexpressing cell line KB-CV60, maintained in medium with 1 μg/mL of cepharanthine and 60 ng/mL of vincristine, were also used in this study (Aoki et al., 2001). HEK293/pcDNA3.1, HEK293/ABCB1, HEK293/ABCG2, and HEK293/ABCC1 cells lines were established by transfecting HEK293 cells with either the empty pcDNA3.1 vector or the vector containing full length ABCB1 (HEK293/ABCB1), ABCG2 (HEK293/ABCG2), and ABCC1 (HEK293/ABCC1) DNA, respectively, and were cultured in medium containing 2 mg/mL of G418 (Enzo Life Sciences, Farmingdale, NY, United States) (Zhang et al., 2015). All cell lines were cultured in DMEM medium with 10% FBS and 1% penicillin/streptomycin at 37°C with 5% CO_2_. All drug-resistant cell lines were grown in a drug-free culture medium for more than 2 weeks prior to use.

### Cell Cytotoxicity by MTT Assay

The cytotoxicity of anticancer drugs with or without modulator agents was determined by modified MTT colorimetric assay (Fan et al., 2018). Briefly, 5000 cells were seeded evenly into each well in coated 96-well microplates overnight. Olmutinib and parallel control modulators were added 2 h prior to the addition of chemotherapeutic drugs in a designated concentration gradient. After 68 h of incubation, 20 μL of MTT solution (4 mg/mL) was added into each well with further incubation of 4 h. The medium was aspirated and 100 μL of DMSO was added to dissolve the formazan crystals in each well. The absorbance was determined at 570 nm by the accuSkan GO UV/Vis Microplate Spectrophotometer (Fisher Scientific, Fair Lawn, NJ, United States). Verapamil, KO-143 and MK-571 was used as a inhibitors for ABCB1-overexpressing, ABCG2-overexpressing, and ABCC1-overexpressing cell lines, respectively.

### Western Blotting Analysis

After treatment with 0, 3, and 6 μM olmutinib for 72 h, and after treatment with 3 μM olmutinib for 24, 48, and 72 h in NCI-H460/MX20 cells, the cells were incubated with a lysis buffer (2.5% 1M Tris, 0.15% EDTA, 1% sodium deoxycholate, 0.1% SDS, 0.88% NaCl, 1% Triton-X and protease inhibitor cocktail) on ice for 20 min, followed by centrifugation at 12,000 *g* at 4°C for 20 min. The supernatant was collected, and the protein concentration was determined by a bicinchoninic acid (BCA)-based protein assay (Thermo Scientific, Rockford, IL, United States). 25 μg of protein in 30 μl loading sample was separated by SDS-polyacrylamide gel electrophoresis and transferred to a polyvinylidene difluoride (PVDF) membrane. After blocked by 5% dry milk for 2 h, the membrane was incubated with primary antibody BXP-34 (1:1000, detects BCRP) overnight at 4°C. The signal was detected using enhanced chemiluminescence followed by incubation with secondary HRP-labeled antibody (1:1000). The protein expression was quantified by ImageJ software (NIH, Bethesda, MD, United States).

### Immunofluorescence Assay

NCI-H460 and NCI-H460/MX20 cells were seeded as 1 × 10^5^ per well in 24-well plates and cultured at 37°C for 24 h, followed by incubation with 3 μM olmutinib for 0, 24, 48, and 72 h, or followed by incubation with 0, 1, 3, or 6 μM olmutinib. The cells were then washed with cold PBS solution twice and fixed in 4% formaldehyde for 15 min. Subsequently, after incubation with 0.5% Triton X-100 for 15 min, cells were incubated with BSA (2 mg/ml) for 1 h followed by monoclonal antibodies for ABCG2 (BXP-34, 1:1000) overnight at 4°C. Cells were further incubated with Alexa Fluor 488 conjugated IgG secondary antibody for 1 h in dark. PI solution was used to counterstain the nuclei. Immunofluorescence images were collected using a Nikon TE-2000S fluorescence microscope (Nikon Instruments Inc., Melville, NY, United States).

### ABCG2 ATPase Assay

The ABCG2 ATPase activity based on vanadate-sensitive membrane vesicles of High Five insect cells was measured as previously described (Zhang et al., 2017). Briefly, the membrane vesicles (10 μg of protein) were incubated in ATPase assay buffer with or without 0.3 mM vanadate at 37°C for 5 min. After that, the assay buffer was incubated with 0–40 μM varying concentrations of olmutinib at 37°C for 3 min. The ATPase reaction was induced by adding 5 mM MgATP with a total volume of 0.1 ml. After 20 min incubation at 37°C, the reaction was stopped by adding 100 μl 5% SDS solution to the reaction mix. The ATPase activity due to ABCG2 is calculated from the amount of inorganic phosphate (IP) released detected at 880 nm using a spectrophotometer.

### Accumulation and Efflux Assay

We used the drug accumulation and efflux assays as previously described (Fan et al., 2018). For the accumulation assay, NCI-H460 and NCI-H460/MX20 cells were seeded into 24-well plate (100,000 cells/well) and incubated at 37°C for 12 h. Then the cells were incubated with or without inhibitors for 2 h. The medium was discarded, followed by the addition of medium containing 0.01 μM [^3^H]-MX, and then the inhibitors were added into the wells. After 2 h incubation, the medium was discarded and the cells were washed with ice-cold PBS three times, lysed, and then transferred to the scintillation fluid. For the efflux assay, we performed similar procedures as the accumulation assay. After discarding the medium containing [^3^H]-MX, the cells were washed with ice-cold PBS and incubated with medium in the absence or presence of inhibitors. The cells were washed three times, lysed, and then transferred to the scintillation fluid at different time points of 0, 30, 60, and 120 min, respectively. The radioactivity was measured using the Packard TRI-CARB1 190‘A liquid scintillation analyzer.

### Molecular Modeling

Molecular modeling was performed in Maestro v11.1 (Schrödinger, LLC) software as described previously (Zhang et al., 2017). Human ABCG2 (PDB ID: 5NJ3) (Taylor et al., 2017) protein preparation was performed and the grid was generated by selecting residues (Phe432, Phe 439, Leu539, Ile543, Val546, and Met549) at a substrate-binding pocket in TMD of ABCG2. The best-scored ligand was obtained through Glide XP docking then the receptor grid for IFD was generated. The IFD protocol with default parameters was performed. The conformation of ligand with the highest docking score (kcal/mol) was used for docking analysis.

### Statistical Analysis

All experiments were repeated at least three times and the result values are presented as mean ± SD. Statistical differences between two groups were determined by the two-tailed Student’s *t*-test and p values equal or below 0.05 were considered significant.

## Results

### Effects of Olmutinib on Cells Overexpressing ABCB1, ABCG2, and ABCC1 Transporters

**FIGURE 2:**
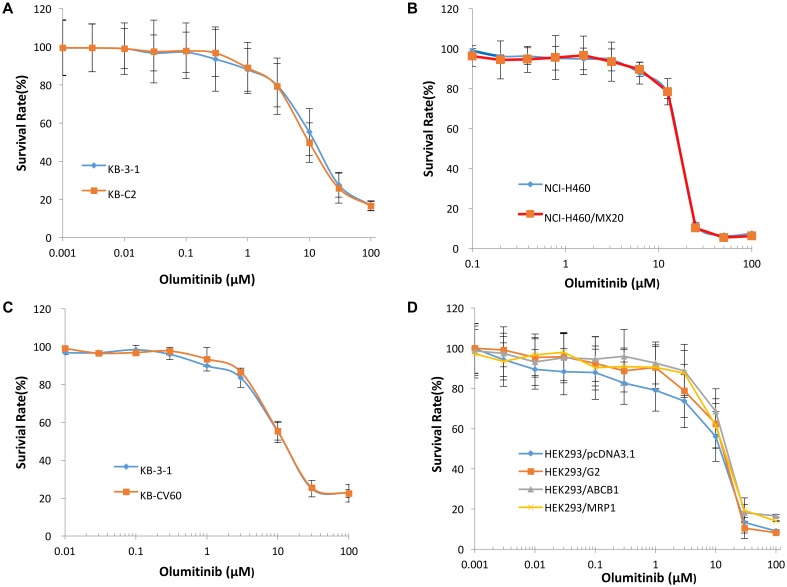
The concentration-survival curve of olmutinib on drug-induced ABCB1-, ABCG2-, and ABCC1-overexpressing cell lines and transfected ABCB1-, ABCG2-, and ABCC1-overexpressing cell lines. **(A)** Concentration-survival curves of KB-3-1 and KB-C2 cell lines incubated only with olmutinib. **(B)** Concentration-survival curves of NCI-H460 and NCI-H460/MX20 cell lines incubated only with olmutinib. **(C)** Concentration-survival curves of KB-3-1 and KB-CV60 cell lines incubated only with olmutinib. **(D)** Concentration-survival curves of HEK293/pcDNA3.1, HEK293/G2, HEK293/ABCB1, and HEK293/MRP1 cell lines incubated only with olmunitib. Each cell line was incubated with a designated concentration gradient of olmunitib for 72 h.

To determine the effects of olmutinib on ABC transporters, the sensitivity of ABCB1-, ABCG2-, and ABCC1-overexpressing cells to olmutinib were determined. The cytotoxicity assay indicated that the IC_50_ values of olmutinib for ABCB1-, ABCG2-, and ABCC1-overexpressing cancer cells (KB-C2, NCI-H460/MX20 and KB-CV60) were 11.42, 12.19, and 17.14 μM. The IC_50_ values of olmutinib for their parental cells (KB-3-1, NCI-H460, and KB-3-1) were 18.85, 15.58, and 12.62 μM, respectively, (**Figure 2**). As the concentration of 3 μM olmutinib did not produce significant cytotoxicity, this concentration was used for reversal experiments.

### Effects of Olmutinib on Reversing Drug-Resistance of ABCG2-Overexpressing Cells

To further determine whether olmutinib in the above cell lines reverses the multi-drug resistance (MDR) mediated by ABC transporters, the drug-induced resistant human cancer cell line (KB-C2, NCI-H460/MX20, and KB-CV60) and the transfected resistant cell line (HEK293/ABCB1, HEK293/G2, and HEK293/MRP1) and their corresponding parental cell lines (KB-3-1, NCI-H460, and HEK293/pcDNA3.1) were used to perform the cytotoxicity assay. As shown in **Tables 1**–**3**, the IC_50_ values of KB-C2 and KB-CV60 cell lines were much higher than those of the corresponding parental cell lines. On the other hand, the result in the NCI-H460/MX20 cell line was the opposite. As compared with the NCI-H460 and HEK293/pcDNA3.1 cell lines, olmutinib significantly decreased the IC_50_ values of MX and SN-38 in the NCI-H460/MX20 and HEK293/ABCG2 cell lines, but it did not affect the values of paclitaxel and vincristine in the KB-C2 and HEK293/ABCB1 cell lines, compared to parental KB-3-1 and HEK293/pcDNA3.1 cell lines. It also did not affect the IC_50_ values of vinblastine and vincristine in KB-CV60 and HEK293/MRP1 cell lines, compared to parental KB-3-1 and HEK293/pcDNA3.1 cell lines. Cisplatin is not a substrate for ABCB1 and ABCG2. It has no reversal effect on ABCB1-mediated and ABCG2-mediated MDR. So there was no significant change in the IC_50_ values of cisplatin in the human cancer cell lines (KB-C2, NCI-H460/MX20, and KB-CV60) or transfected cell lines (HEK293/ABCB1, HEK293/G2, and HEK293/MRP1) compared with the corresponding parental cells (**Tables 1**–**3**). These results indicate that olmutinib could selectively reverse MDR mediated by ABCG2-overexpression but could not reverse ABCB1- and ABCC1-overexpressing mediated MDR.

**Table 1:** The effect of olmutinib on drug resistance to paclitaxel, vincristine, and cisplatin in ABCB1-overexpressing cell lines (KB-3-1 and KB-C2, HEK293/pcDNA3.1, and HEK293/ABCB1).

Treatment	IC_50_ ± SD^a^ (RF^b^)			
	KB-3-1 (μM)	KB-C2 (μM)	HEK293/pcDNA3.1 (μM)	HEK293/ABCB1 (μM)
**Paclitaxel**	0.034 ± 0.007(1.00)	2.389 ± 0.541(70.26)	0.173 ± 0.072(1.00)	4.464 ± 0.466(25.80)
+ Olmutinib (1 μM)	0.038 ± 0.009(1.12)	2.237 ± 0.487(65.79)	0.158 ± 0.025(0.91)	3.653 ± 1.096(21.12)
+ Olmutinib (3 μM)	0.036 ± 0.007(1.06)	2.174 ± 0.641(63.94)	0.134 ± 0.074(0.77)	3.462 ± 0.981(20.01)
+ Verapamil (3 μM)	0.054 ± 0.016(1.59)	0.495 ± 0.144(14.56)^∗∗^	0.153 ± 0.146(0.88)	0.263 ± 0.034(1.52)^∗∗^
**Vincristine**	0.028 ± 0.006(1.00)	1.299 ± 0.361(46.39)	0.086 ± 0.221(1.00)	0.763 ± 0.647(8.87)
+ Olmutinib (1 μM)	0.024 ± 0.006(0.86)	1.217 ± 0.389(43.46)	0.071 ± 0.016(0.83)	0.733 ± 0.068(8.52)
+ Olmutinib (3 μM)	0.021 ± 0.007(0.75)	1.132 ± 0.238(40.43)	0.063 ± 0.017(0.73)	0.651 ± 0.692 (7.57)
+ Verapamil (3 μM)	0.032 ± 0.015(1.14)	0.056 ± 0.011(2.00)^∗∗^	0.077 ± 0.013(0.90)	0.126 ± 0.051(1.47)
**Cisplatin**	1.194 ± 0.876(1.00)	1.863 ± 0.428(1.56)	1.523 ± 0.285(1.00)	1.945 ± 0.876(1.28)
+ Olmutinib (3 μM)	1.123 ± 0.473(0.94)	1.346 ± 0.283(1.13)	1.433 ± 0.465(0.94)	1.683 ± 0.464(1.11)
+ Verapamil (3 μM)	1.744 ± 0.452(1.46)	1.643 ± 0.233(1.38)	1.245 ± 0.337(0.82)	1.534 ± 0.344(1.01)

*^a^IC_50_ values represent the mean ± SD of three independent experiments which were performed in triplicate. ^b^Resistance Fold (RF) was calculated by dividing the IC_50_ values of substrates in the presence or absence of an inhibitor by the IC_50_ values of parental cells without an inhibitor. ^∗∗^P < 0.01 versus no inhibitor group.*

**Table 2:** The effect of olmutinib on drug resistance to MX, SN38, and cisplatin in ABCG2-overexpressing cell lines (NCI-H460 and NCI-H460/MX20, and HEK293/pcDNA3.1 and HEK293/ABCG2).

Treatment	IC_50_ ± SD^a^ (RF^b^)			
	NCI-H460 (nM)	NCI-H460/MX20 (μM)	HEK293/pcDNA3.1 (nM)	HEK293/ABCG2 (μM)
**MX**	22.987 ± 4.145(1.00)	2.327 ± 0.642(101.23)	72.245 ± 10.834(1.00)	1.974 ± 0.846(27.32)
+ Olmutinib (1 μM)	18.642 ± 4.101(0.81)	0.039 ± 0.009(1.70)^∗∗^	70.563 ± 20.169(0.98)	0.261 ± 0.052(3.61)^∗^
+ Olmutinib (3 μM)	17.067 ± 2.241(0.74)	0.027 ± 0.017(1.17)^∗∗^	68.125 ± 14.824(0.94)	0.146 0.038(2.02)^∗^
+ Ko 143 (3 μM)	16.947 ± 2.643(0.74)	0.022 ± 0.012(0.96)^∗∗^	62.234 ± 11.216(0.86)	0.183 ± 0.022(2.53)^∗^
**SN-38**	13.454 ± 1.156(1.00)	2.197 ± 0.342(163.30)	56.462 ± 9.243(1.00)	2.389 ± 0.279(42.31)
+ Olmutinib (1 μM)	12.592 ± 2.392(0.94)	0.033 ± 0.012(2.45)^∗∗^	56.427 ± 10.421(1.00)	0.288 ± 0.038(5.10)^∗∗^
+ Olmutinib (3 μM)	12.346 ± 3.243(0.92)	0.019 ± 0.034(1.41)^∗∗^	54.364 ± 5.826(0.96)	0.176 ± 0.029(3.12)^∗∗^
+ Ko 143 (3 μM)	12.456 ± 4.024(0.93)	0.021 ± 0.032(1.56)^∗∗^	57.522 ± 8.024(1.02)	0.164 ± 0.027(2.90)^∗∗^

**IC_50_ ± SD^a^ (μM) (RF^b^)**				

**Cisplatin**	1.426 ± 0.543(1.00)	1.745 ± 0.224(1.22)	1.628 ± 0.252(1.00)	1.463 ± 0.663(0.90)
+ Olmutinib (3 μM)	1.313 ± 0.512(0.92)	1.654 ± 0.443(1.16)	1.214 ± 0.478(0.75)	1.126 ± 0.275(0.69)
+ Ko 143 (3 μM)	1.226 ± 0.861(0.86)	1.494 ± 0.268(1.05)	1.354 ± 0.368(0.83)	1.425 ± 0.264(0.88)

*^a^IC_50_ values represent the mean ± SD of three independent experiments which were performed in triplicate. ^b^Resistance Fold (RF) was calculated by dividing the IC_50_ values of substrates in the presence or absence of an inhibitor by the IC_50_ values of parental cells without an inhibitor. ^∗^P < 0.05, ^∗∗^P < 0.01 versus no inhibitor group.*

**Table 3:** The effect of olmutinib on drug resistance to vincristine, vinblastine, and cisplatin in ABCC1-overexpressing cell lines (KB-3-1and KB-CV60, HEK293/pcDNA3.1, and HEK293/ABCC1).

Treatment	IC_50_ ± SD^a^ (RF^b^)			
	KB-3-1 (nM)	KB-CV60 (nM)	HEK293/pcDNA3.1 (nM)	HEK293/ABCC1 (nM)
**Vincristine**	18.424 ± 1.783(1.00)	253.243 ± 11.45(13.75)	13.254 ± 1.884(1.00)	258.344 ± 16.76(19.49)
+ Olmutinib (1 μM)	17.019 ± 2.354(0.92)	230.565 ± 14.081(12.51)	13.136 ± 2.372(0.99)	221.357 ± 20.731(16.70)
+ Olmutinib (3 μM)	16.564 ± 2.165(0.90)	224.363 ± 13.47(12.18)	12.136 ± 2.565(0.92)	213.564 ± 18.672(16.11)
+ MK 571 (25 μM)	17.434 ± 1.624(0.95)	22.467 ± 9.744(1.22)^∗∗^	11.457 ± 1.343(0.86)	45.348 ± 7.238(3.42)^∗∗^
**Vinblastine**	56.514 ± 9.584(1.00)	275.345 ± 13.545(4.87)	8.864 ± 0.843(1.00)	58.462 ± 6.453(6.60)
+ Olmutinib (1 μM)	56.235 ± 7.956(1.00)	234.864 ± 18.452(4.16)	8.233 ± 0.571(0.93)	46.892 ± 7.563(5.29)
+ Olmutinib (3 μM)	56.184 ± 4.763(0.99)	213.585 ± 10.842(3.80)	8.164 ± 0.263(0.92)	45.364 ± 6.837(5.12)
+ MK 571 (25 μM)	45.527 ± 6.254(0.81)	75.613 ± 8.124(1.34)^∗∗^	7.852 ± 0.144(0.89)	9.823 ± 0.453(1.11)^∗∗^

**IC_50_ ± SD^a^ (μM) (RF^b^)**				

**Cisplatin**	1.683 ± 0.562(1.00)	1.896 ± 0.323(1.13)	1.644 ± 0.253(1.00)	1.864 ± 0.362(1.13)
+ Olmutinib (3 μM)	1.724 ± 0.233(1.02)	1.657 ± 0.546(0.98)	1.775 ± 0.265(1.08)	1.626 ± 0.164(0.99)
+MK 571 (25 μM)	1.326 ± 0.364(0.79)	1.469 ± 0.328(0.87)	1.527 ± 0.263(0.93)	1.454 ± 0.246(0.88)

*^a^IC_50_ values represent the mean ± SD of three independent experiments which were performed in triplicate. ^b^Resistance Fold (RF) was calculated by dividing the IC_50_ values of substrates in the presence or absence of an inhibitor by the IC_50_ values of parental cells without an inhibitor. ^∗∗^P < 0.01 versus no inhibitor group.*

### Effect of Olmutinib on the Protein Expression of ABCG2 Transporters

**FIGURE 3:**
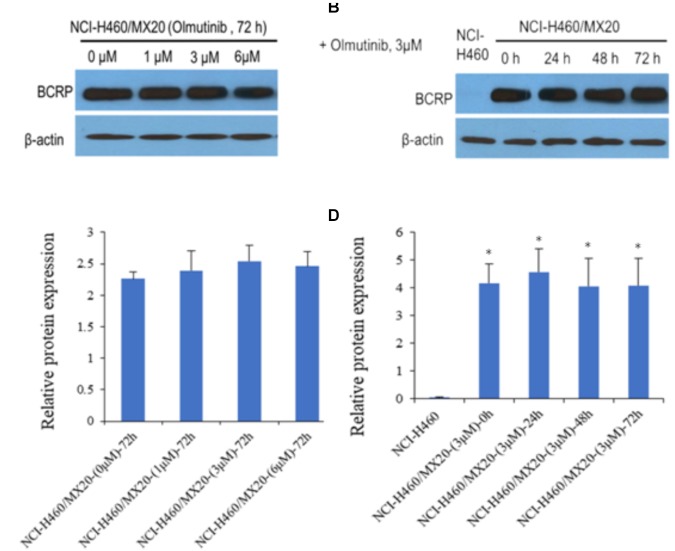
Western blotting to detect ABCG2 expression in ABCG2-over-expressing cell lines. **(A)** The effect of olmutinib at 1, 3, and 6 μM on the expression levels of ABCG2 in NCI-H460 and NCI-H460/MX20 cells at 72 h. **(B)** The effect of olmutinib at 3 μM on the expression levels of ABCG2 in NCI-H460 and NCI-H460/MX20 cells at 0, 24, 48, and 72 h. **(C)** The effect of olmutinib at 0, 1, 3, and 6 μM on the expression levels of ABCG2 in NCI-H460 and NCI-H460/MX20 cells at 72 h. **(D)** The effect of olmutinib at 3 μM on the expression levels of ABCB1 in NCI-H460 and NCI-H460/MX20 cells at 0, 24, 48, and 72 h. Equal amounts of total cell lysate were used for each sample. ^∗^*P* < 0.05 versus the control group (**B,D** versus NCI-H460).

Western blot analysis was performed to confirm whether olmutinib could affect the protein expression of ABCG2 transporters in cell lysates. As shown in **Figures 3A,C**, different concentrations (1, 3, and 6 μM) of olmutinib treatment did not significantly alter the expression of the ABCG2 protein (72 kDa) in ABCG2-overexpressing NCI-H460/MX20 cell line compared to control. **Figures 3B,D** indicated that the protein expression level of ABCG2 was quite low in the parental NCI-H460 cell line, and the treatment of olmutinib (3 μM) for 0, 24, 48, and 72 h did not significantly alter expression of the ABCG2 protein (72 kDa) in the ABCG2-overexpressing NCI-H460/MX20 cell line.

### Effect of Olmutinib on the Expression and Intracellular Localization of ABCG2

**FIGURE 4:**
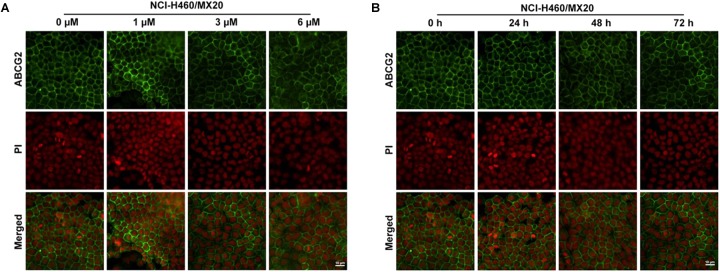
Effect of olmutinib on the expression and cell localization of ABCG2. **(A)** Micrographs are representatives of immunostaining of NCI-H460/MX20 cells incubated with 3 mM olmutinib after 0, 24, 48, and 72 h. **(B)** The effect of olmutinib at 0, 1, 3, and 6 mM on NCI-H460/MX20 cells after being processed for 72 h.

To further confirm whether olmutinib altered the expression and cellular localization of the ABCG2 protein, immunofluorescence staining was performed after cells were processed with different time of incubation or with different concentrations of olmutinib. As shown in **Figure 4**, the ABCG2 transporters are located on the membrane of NCI-H460/MX20 cells. **Figure 4A** showed that incubation of cells with 3 μM of olmutinib did not significantly alter the subcellular distribution of ABCG2 in NCI-H460/MX20 cells when compared at 0, 24, 48, and 72 h. Similarly, **Figure 4B** showed that incubation of cells after 72 h with 0, 1, 3, and 6 μM of olmutinib did not significantly alter the subcellular distribution of ABCG2 in NCI-H460/MX20 cells.

### Effect of Olmutinib on [^3^H]-MX Accumulation and Efflux

**FIGURE 5:**
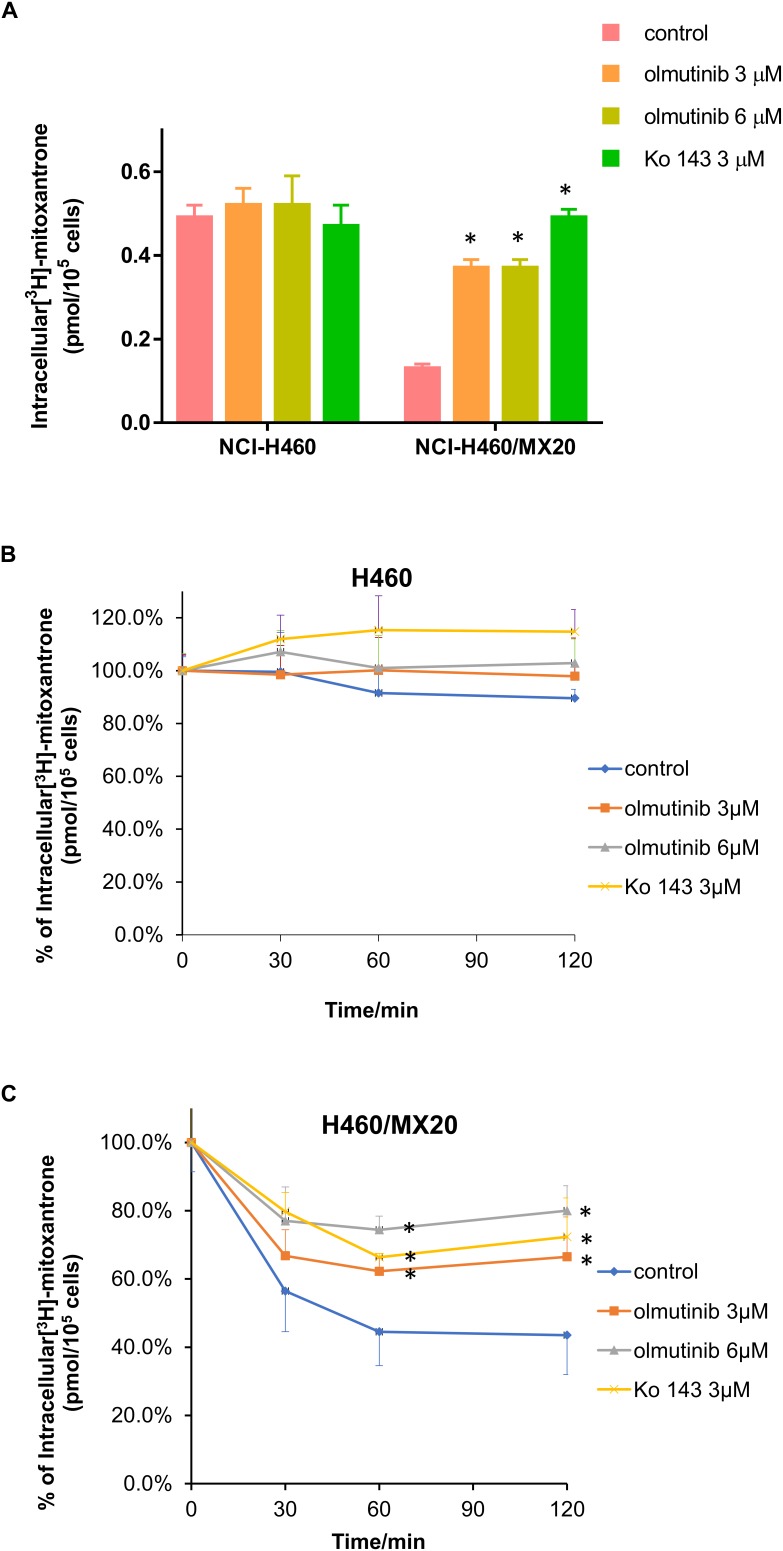
Effects of olmutinib on intracellular accumulation and efflux of [^3^H]-MX in NCI-H460 and NCI-H460/MX20 cells. **(A)** The effects of olmutinib on the accumulation of [^3^H]-MX in NCI-H460 and NCI-H460/MX20 cells. **(B)** The effects of olmutinib on the efflux function of NCI-H460 cells. **(C)** The effects of olmutinib on the efflux function of NCI-H460/MX20 cells. Error bars represent the SD value. Ko 143 (3 μM) is used as positive control for ABCG2-overexpressing cells. ^∗^*P* < 0.05 versus control group, two-way ANOVA.

The accumulation and efflux effects of the olmutinib were investigated by comparing the quantity of [^3^H]-MX in NCI-H460 and ABCG2-mediated NCI-H460/MX20 cells. As shown in **Figure 5A**, intracellular [^3^H]-MX level in NCI-H460/MX20 cells was approximately 2.5-fold lower than that in NCI-H460 cells after 2 h incubation without an inhibitor. Compared with the control group, the accumulation of [^3^H]-MX in NCI-H460/MX20 cells was significantly increased in the olmutinib (3 μM) group. The accumulation effect of olmutinib (3 μM) is comparable to that of Ko 143 (3 μM), which is a positive inhibitor of ABCG2. **Figure 5B** indicated that the intracellular [^3^H]-MX level in NCI-H460 cells did not significantly change after 120 min, and treatment with inhibitors did not alter the efflux function in NCI-H460 cells. However, the intracellular [^3^H]-MX level in NCI-H460/MX20 cells decreased dramatically by about 60% without the treatment of inhibitors (**Figure 5C**). With the treatment of olmutinib (3 μM), the efflux function of NCI-H460/MX20 cells could be effectively inhibited; this inhibition of efflux function would increase with olmutinib at 6 μM.

### Effect of Olmutinib on ATPase Activity of ABCG2 Transporters

**FIGURE 6:**
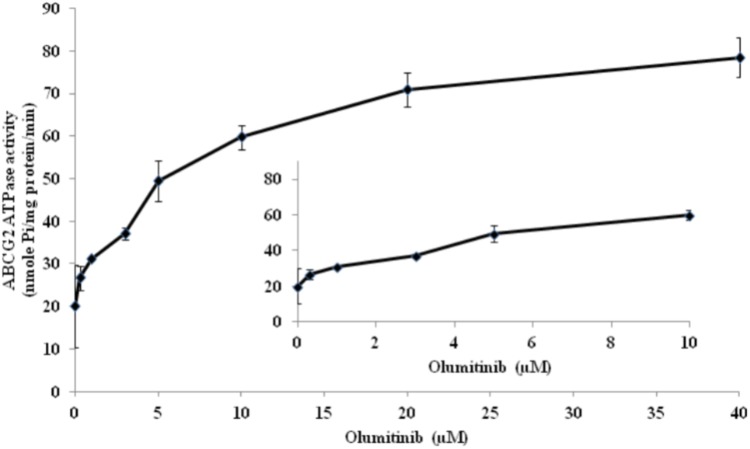
ABCG2 transporter specific ATPase activity was stimulated by olmutinib. The effect of olmutinib on the subcellular localization of BCRP in NCI-H460/MX20 cells. The graph shows that ATPase activity was plotted with SD as a function of concentration of olmutinib. The inset shows stimulation of ATP hydrolysis at lower (0–10 μM) concentration of olmutinib.

ABCG2-mediated ATP hydrolysis, in the presence of olmutinib at various concentrations from 0 to 40 μM, was measured to assess the effect of olmutinib on the ATPase activity of ABCG2. Olmutinib stimulated the ATPase activity of ABCG2 in a concentration-dependent manner, with a maximal stimulation of 3.5-fold of the basal activity as shown in **Figure 6**. The concentration of olmutinib required to obtain 50% stimulation is 2.2 μM. This result indicated that olmutinib interacts at the drug-substrate binding site and affects the ATPase activity of ABCG2.

### Docking Analysis of the Binding of Olmutinib With Human Wild-Type ABCG2 Model

**FIGURE 7:**
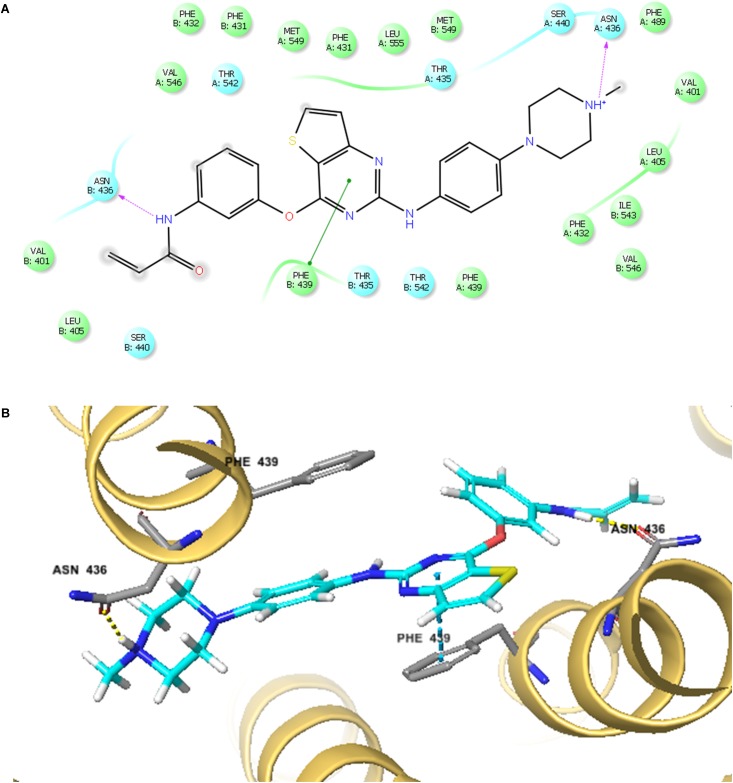
The docking simulation of olmutinib with human ABCG2. **(A)** The two-dimensional ligand–receptor interaction diagram of olmutinib and human ABCG2. The amino acids within 4 Å are shown as colored bubbles, cyan indicates polar residues, and green indicates hydrophobic residues. Hydrogen bonds are shown by the purple dotted arrow. **(B)** Docked position of olmutinib within the binding site of human ABCG2. Olmutinib is shown as ball and stick mode with the atoms colored: carbon – cyan, hydrogen – white, nitrogen – blue, oxygen – red, and sulfur – yellow. Important residues are shown as sticks with the same color mode as above except that carbons are colored in gray.

The best-scored docked (-10.902 kcal/mol) position of olmutinib within the binding pocket of human ABCG2 (5NJ3) is shown in **Figure 7**. **Figure 7A** showed that there were two hydrogen bonds and one π – π interaction between olmutinib and human ABCG2. Interestingly, the nitrogen in piperazine group of olmutinib was ionized and formed a hydrogen bond with Asn436 in the A chain. It was reported that acidic microenvironment is a major feature of tumor (Kato et al., 2013). Acidic extracellular pH could facilitate the ionization of olmutinib and generate the hydrogen bonding with ABCG2. The nitrogen in the enamide group of olmutinib could have another hydrogen bonding with Asn436 in the B chain of ABCG2. Besides the interactions mentioned above, olmutinib could be stabilized in the hydrophobic pocket of ABCG2 by the hydrophobic interactions with residues such as Met431, Phe432, Ile543, Phe549, and Leu555.

## Discussion

According to previous studies, small molecule cell signaling inhibitors, such as TK inhibitors, are of crucial importance in chemotherapeutic drug resistance (Wu et al., 2014). ABC transporters are known as mediators of MDR, which is significantly reversed by many clinically used TKIs. Cancer cells acquire MDR via various types of ABC transporters; for example NSCLC cells obtain MDR due to overexpression of ABCG2. ABC transporter-mediated MDR has been found partially or completely reversed by other TKIs such as fumitremorgin C (FTC) (Kim, 2016). As a third-generation EGFR TKI, olmutinib has been approved for the treatment of locally advanced or metastatic EGFR T790M mutation-positive NSCLC (Ni et al., 2010). Therefore, in this study, we aimed to further explore whether olmutinib has potential reversal effects in different types of ABC transporter-mediated MDR. The results indicated that olmutinib greatly reversed ABCG2-mediated MDR, while ABCB1- and ABCC1-mediated MDR were not significantly influenced.

According to our MTT assay, MX-selected NCI-H460/MX20 cells also acquired resistance to SN-38 and were sensitized to both MX and SN-38 by incubation with olmutinib. IC_50_ of cisplatin, which is not a substrate of ABCG2, did not vary significantly with or without pre-incubation with olmutinib. To further explore the specificity of its reversal effects, we also determined the effects of olmutinib on ABCB1-overexprssing cells. Both ABCB1-transfected HEK293 cell and colchicine-selected ABCB1-overexpressing KB-C2 cells had shown drug resistance toward paclitaxel and vincristine. After olmutinib incubation, the results showed that ABCB1-overexpressing cells were not significantly sensitized. Similarly, olmutinib did not show statistically significant reversal effects in ABCC1-overexpressing KB-CV60 and HEK293/ABCC1 cells.

Moreover, to explore the possible mechanism of olmutinib, its effect on ABCG2 expression was tested. Based on the result, ABCG2 expression was not significantly altered with different incubation time or olmutinib concentration. Additionally, intracellular localization of ABCG2 did not vary after 72 h of treatment, as demonstrated by the immunofluorescence results. Based on these findings, we can conclude that the reversal of drug resistance by olmutinib is not related to down-regulated ABCG2 protein expression or altered intracellular location. Instead, the reversal function of olmutinib may be generated by interacting with the ABCG2 protein.

To further determine the interaction between olmutinib and ABCG2, we investigated the accumulation/efflux effect of olmutinib. The results indicated a significant increase in [^3^H]-MX accumulation in NCI-H460MX20 cells when incubated with 3 μM olmutinib. The accumulation effect of olmutinib was comparable to that of a positive ABCG2 inhibitor Ko 143. In addition, according to the efflux time-course, MDR cells showed an inhibited MX efflux when treated with olmutinib compared with control group. In conclusion, olmutinib reversed ABCG2-mediated MDR due to inhibited drug efflux, leading to an increased intracellular drug accumulation. A large proportion of TKIs that are involved in ABC transporter-mediated MDR reversal stimulate ATP hydrolysis (Anreddy et al., 2014); we saw consistent results in the ATPase assay that olmutinib stimulated ATPase activity of ABCG2 up to 3.5-fold. Additionally, the molecular interaction between olmutinib and ABCG2 was identified by a docking simulation. The best-score (-10.902 kcal/mol) docking position of olmutinib within human ABCG2 implied direct interaction with π – π bonds and hydrogen bonds, which stabilized olmutinib in the hydrophobic pocket of ABCG2. The results showed that olmutinib not only interacts directly with ABCG2 but also works as a competitive inhibitor of the transport protein. In conclusion, the reversal effect of olmutinib on ABCG2 in MDR cells is not due to ABCG2 expression or intracellular localization, but is related to its interaction with the ABCG2 protein including drug efflux inhibition and ATPase stimulation.

The mechanism involved in resistance of olmutinib is very complex. In recent years, some studies have shown that the nucleotide polymorphism of ABCG2 gene may play a role in the expression level and function of ABCG2 (Tanaka et al., 2011; Chen et al., 2015; Tang et al., 2018). In addition, the nucleotide polymorphism of ABCG2 gene is also associated with drug resistance. Therefore, the effect of ABCG2 single nucleotide polymorphism on ABCG2 function, pharmacokinetics and the mechanism of ABCG2 effect on tumor will be the focus of our future research.

## Conclusion

Overall, in this study we reported that a newly approved TKI olmutinib reversed ABCG2-mediated MDR by inhibiting chemotherapeutic drug efflux and increasing intracellular drug accumulation. Collectively, our study suggested that ABCG2-mediated drug resistance to conventional chemotherapeutic drugs could be reversed by a combination regimen of olmutinib with other chemotherapeutic drugs.

## Author Contributions

Z-SC conceived and designed the experiments. WZ, Y-FF, C-YC, J-QW, Q-XT, Z-NL, LZ, and PG performed the experiments and wrote the manuscript. Z-SC finalized the manuscript. All authors read and approved the manuscript.

## Conflict of Interest Statement

The authors declare that the research was conducted in the absence of any commercial or financial relationships that could be construed as a potential conflict of interest.

## Abbreviations

ABC: ATP-binding cassette
EGFR: epidermal growth factor receptor
IFD: induced-fit docking
MDR: multidrug resistance
MTT: 3-(4, 5- dimethylthiazol-yl)-2, 5- diphenyllapatinibrazolium bromide
MX: mitoxantrone
TK: tyrosine kinase
TKI: tyrosine kinase inhibitor
TMD: transmembrane domain.

## References

[B1] Anreddy N. Gupta P. Kathawala R. J. Patel A. Wurpel J. N. Chen Z. S. (2014). Tyrosine kinase inhibitors as reversal agents for ABC transporter mediated drug resistance. *Molecules* 19 13848–13877. 10.3390/molecules190913848 25191874 PMC6271846

[B2] Aoki S. Chen Z. S. Higasiyama K. Setiawan A. Akiyama S. Kobayashi M. (2001). Reversing effect of agosterol A, a spongean sterol acetate, on multidrug resistance in human carcinoma cells. *Jpn. J. Cancer. Res.* 92 886–895. 10.1111/j.1349-7006.2001.tb01177.x 11509122 PMC5926837

[B3] Beretta G. L. Cassinelli G. Pennati M. Zuco V. Gatti L. (2017). Overcoming ABC transporter-mediated multidrug resistance: the dual role of tyrosine kinase inhibitors as multitargeting agents. *Eur. J. Med. Chem.* 142 271–289. 10.1016/j.ejmech.2017.07.062 28851502

[B4] Borst P. Kool M. Evers R. (1997). Do cMOAT (MRP2), other MRP homologues, and LRP play a role in MDR? *Semin. Cancer Biol.* 8 205–213. 10.1006/scbi.1997.0071 9441949

[B5] Chen X. Chen D. Yang S. Ma R. Pan Y. Li X. (2015). Impact of ABCG2 polymorphisms on the clinical outcome of TKIs therapy in Chinese advanced non-small-cell lung cancer patients. *Cancer Cell Int.* 15 43. 10.1186/s12935-015-0191-3 25960692 PMC4425882

[B6] Chen Y. Zhang H. Zhang Y. (2017). Targeting receptor tyrosine kinase EphB4 in cancer therapy. *Semin. Cancer Biol.* 10.1016/j.semcancer.2017.10.002 [Epub ahead of print]. 28993206

[B7] Chikhale R. Thorat S. Choudhary R. K. Gadewal N. Khedekar P. (2018). Design, synthesis and anticancer studies of novel aminobenzazolyl pyrimidines as tyrosine kinase inhibitors. *Bioorg. Chem.* 77 84–100. 10.1016/j.bioorg.2018.01.008 29342447

[B8] Cole S. P. Bhardwaj G. Gerlach J. H. Mackie J. E. Grant C. E. Almquist K. C. (1992). Overexpression of a transporter gene in a multidrug-resistant human lung cancer cell line. *Science* 258 1650–1654. 10.1126/science.1360704 1360704

[B9] Dohse M. Scharenberg C. Shukla S. Robey R. W. Volkmann T. Deeken J. F. (2010). Comparison of ATP-binding cassette transporter interactions with the tyrosine kinase inhibitors imatinib, nilotinib, and dasatinib. *Drug Metab. Dispos.* 38 1371–1380. 10.1124/dmd.109.031302 20423956 PMC2913625

[B10] Doyle L. A. Yang W. Abruzzo L. V. Krogmann T. Gao Y. Rishi A. K. (1998). A multidrug resistance transporter from human MCF-7 breast cancer cells. *Proc. Natl. Acad. Sci. U.S.A.* 95 15665–15670. 10.1073/pnas.95.26.15665 9861027 PMC28101

[B11] Faivre S. Djelloul S. Raymond E. (2006). New paradigms in anticancer therapy: targeting multiple signaling pathways with kinase inhibitors. *Semin. Oncol.* 33 407–420. 10.1053/j.seminoncol.2006.04.005 16890796

[B12] Fan Y. F. Zhang W. Zeng L. Lei Z. N. Cai C. Y. Gupta P. (2018). Dacomitinib antagonizes multidrug resistance (MDR) in cancer cells by inhibiting the efflux activity of ABCB1 and ABCG2 transporters. *Cancer Lett.* 421 186–198. 10.1016/j.canlet.2018.01.021 29331420

[B13] Fletcher J. I. Williams R. T. Henderson M. J. Norris M. D. Haber M. (2016). ABC transporters as mediators of drug resistance and contributors to cancer cell biology. *Drug Resist. Updat.* 26 1–9. 10.1016/j.drup.2016.03.001 27180306

[B14] Gillet J. P. Gottesman M. M. (2010). Mechanisms of multidrug resistance in cancer. *Methods Mol. Biol.* 596 47–76. 10.1007/978-1-60761-416-6-4 19949920

[B15] Herbrink M. Nuijen B. Schellens J. H. Beijnen J. H. (2015). Variability in bioavailability of small molecular tyrosine kinase inhibitors. *Cancer Treat. Rev.* 41 412–422. 10.1016/j.ctrv.2015.03.005 25818541

[B16] Hocker S. E. Higginbotham M. L. Schermerhorn T. Henningson J. (2017). Receptor tyrosine kinase expression and phosphorylation in canine nasal carcinoma. *Res. Vet. Sci.* 115 484–489. 10.1016/j.rvsc.2017.07.030 28783596

[B17] Hu T. Li Z. Gao C. Y. Cho C. H. (2016). Mechanisms of drug resistance in colon cancer and its therapeutic strategies. *World J. Gastroenterol.* 22 6876–6889. 10.3748/wjg.v22.i30.6876 27570424 PMC4974586

[B18] Juliano R. L. Ling V. (1976). A surface glycoprotein modulating drug permeability in Chinese hamster ovary cell mutants. *Biochim. Biophys. Acta* 455 152–162. 10.1016/0005-2736(76)90160-7 990323

[B19] Kartal-Yandim M. Adan-Gokbulut A. Baran Y. (2016). Molecular mechanisms of drug resistance and its reversal in cancer. *Crit. Rev. Biotechnol.* 36 716–726. 10.3109/07388551.2015.1015957 25757878

[B20] Kartner N. Riordan J. R. Ling V. (1983). Cell surface P-glycoprotein associated with multidrug resistance in mammalian cell lines. *Science* 221 1285–1288. 10.1126/science.6137059 6137059

[B21] Kathawala R. J. Li T. Yang D. Guo H. Q. Yang D. H. Chen X. (2017). 2-trifluoromethyl-2-hydroxypropionamide derivatives as novel reversal agents of ABCG2 (BCRP)-mediated multidrug resistance: synthesis and biological evaluations. *J. Cell. Biochem.* 118 2420–2429. 10.1002/jcb.25908 28120346 PMC5462856

[B22] Kato Y. Ozawa S. Miyamoto C. Maehata Y. Suzuki A. Maeda T. (2013). Acidic extracellular microenvironment and cancer. *Cancer Cell Int.* 13:89. 10.1186/1475-2867-13-89 24004445 PMC3849184

[B23] Kim E. S. (2016). Olmutinib: first global approval. *Drugs* 76 1153–1157. 10.1007/s40265-016-0606-z 27357069

[B24] Krishna R. Mayer L. D. (2000). Multidrug resistance (MDR) in cancer. Mechanisms, reversal using modulators of MDR and the role of MDR modulators in influencing the pharmacokinetics of anticancer drugs. *Eur. J. Pharm. Sci.* 11 265–283. 10.1016/S0928-0987(00)00114-7 11033070

[B25] Ma S. L. Hu Y. P. Wang F. Huang Z. C. Chen Y. F. Wang X. K. (2014). Lapatinib antagonizes multidrug resistance-associated protein 1-mediated multidrug resistance by inhibiting its transport function. *Mol. Med.* 20 390–399. 10.2119/molmed.2014.00059 25105301 PMC4212010

[B26] Miklos W. Pelivan K. Kowol C. R. Pirker C. Dornetshuber-Fleiss R. Spitzwieser M. (2015). Triapine-mediated ABCB1 induction via PKC induces widespread therapy unresponsiveness but is not underlying acquired triapine resistance. *Cancer Lett.* 361 112–120. 10.1016/j.canlet.2015.02.049 25749419

[B27] Miyake K. Mickley L. Litman T. Zhan Z. Robey R. Cristensen B. (1999). Molecular cloning of cDNAs which are highly overexpressed in mitoxantrone-resistant cells: demonstration of homology to ABC transport genes. *Cancer Res.* 59 8–13. 9892175

[B28] Ni Z. Bikadi Z. Rosenberg M. F. Mao Q. (2010). Structure and function of the human breast cancer resistance protein (BCRP/ABCG2). *Curr. Drug Metab.* 11 603–617. 10.2174/138920010792927325 20812902 PMC2950214

[B29] Pan S. T. Li Z. L. He Z. X. Qiu J. X. Zhou S. F. (2016). Molecular mechanisms for tumour resistance to chemotherapy. *Clin. Exp. Pharmacol. Physiol.* 43 723–737. 10.1111/1440-1681.12581 27097837

[B30] Parikh P. K. Ghate M. D. (2018). Recent advances in the discovery of small molecule c-Met kinase inhibitors. *Eur. J. Med. Chem.* 143 1103–1138. 10.1016/j.ejmech.2017.08.044 29157685

[B31] Pytel D. Sliwinski T. Poplawski T. Ferriola D. Majsterek I. (2009). Tyrosine kinase blockers: new hope for successful cancer therapy. *Anticancer Agents Med. Chem.* 9 66–76. 10.2174/187152009787047752 19149483

[B32] Robey R. W. Pluchino K. M. Hall M. D. Fojo A. T. Bates S. E. Gottesman M. M. (2018). Revisiting the role of ABC transporters in multidrug-resistant cancer. *Nat. Rev. Cancer* 18 452–464. 10.1038/s41568-018-0005-8 29643473 PMC6622180

[B33] Robey R. W. Polgar O. Deeken J. To K. W. Bates S. E. (2007). ABCG2: determining its relevance in clinical drug resistance. *Cancer Metastasis Rev.* 26 39–57. 10.1007/s10555-007-9042-6 17323127

[B34] Russo A. Franchina T. Ricciardi G. R. R. Smiroldo V. Picciotto M. Zanghi M. (2017). Third generation EGFR TKIs in EGFR-mutated NSCLC: where are we now and where are we going. *Crit. Rev. Oncol. Hematol.* 117 38–47. 10.1016/j.critrevonc.2017.07.003 28807234

[B35] Saraswathy M. Gong S. (2013). Different strategies to overcome multidrug resistance in cancer. *Biotechnol. Adv.* 31 1397–1407. 10.1016/j.biotechadv.2013.06.004 23800690

[B36] Shukla S. Chen Z. S. Ambudkar S. V. (2012). Tyrosine kinase inhibitors as modulators of ABC transporter-mediated drug resistance. *Drug Resist. Updat.* 15 70–80. 10.1016/j.drup.2012.01.005 22325423 PMC3348341

[B37] Singh M. Jadhav H. R. (2018). Targeting non-small cell lung cancer with small-molecule EGFR tyrosine kinase inhibitors. *Drug Discov. Today* 23 745–753. 10.1016/j.drudis.2017.10.004 29031620

[B38] Strouse J. J. Ivnitski-Steele I. Waller A. Young S. M. Perez D. Evangelisti A. M. (2013). Fluorescent substrates for flow cytometric evaluation of efflux inhibition in ABCB1, ABCC1, and ABCG2 transporters. *Anal. Biochem.* 437 77–87. 10.1016/j.ab.2013.02.018 23470221 PMC3785545

[B39] Tanaka M. Okazaki T. Suzuki H. Abbruzzese J. L. Li D. (2011). Association of multi-drug resistance gene polymorphisms with pancreatic cancer outcome. *Cancer* 117 744–751. 10.1002/cncr.25510 20922799 PMC3017663

[B40] Tang L. Zhang C. He H. Pan Z. Fan D. He Y. (2018). Associations between ABCG2 gene polymorphisms and gefitinib toxicity in non-small cell lung cancer: a meta-analysis. *Onco Targets Ther.* 11 665–675. 10.2147/OTT.S154244 29440914 PMC5798561

[B41] Taylor N. M. I. Manolaridis I. Jackson S. M. Kowal J. Stahlberg H. Locher K. P. (2017). Structure of the human multidrug transporter ABCG2. *Nature* 546 504–509. 10.1038/nature22345 28554189

[B42] van der Geer P. Hunter T. Lindberg R. A. (1994). Receptor protein-tyrosine kinases and their signal transduction pathways. *Annu. Rev. Cell Biol.* 10 251–337. 10.1146/annurev.cb.10.110194.001343 7888178

[B43] Wu Q. Yang Z. Nie Y. Shi Y. Fan D. (2014). Multi-drug resistance in cancer chemotherapeutics: mechanisms and lab approaches. *Cancer Lett.* 347 159–166. 10.1016/j.canlet.2014.03.013 24657660

[B44] Zhang X. Y. Zhang Y. K. Wang Y. J. Gupta P. Zeng L. Xu M. (2016a). Osimertinib (AZD9291), a mutant-selective EGFR inhibitor, reverses ABCB1-mediated drug resistance in cancer cells. *Molecules* 21:E1236. 10.3390/molecules21091236 27649127 PMC6273565

[B45] Zhang Y. K. Zhang G. N. Wang Y. J. Patel B. A. Talele T. T. Yang D. H. (2016b). Bafetinib (INNO-406) reverses multidrug resistance by inhibiting the efflux function of ABCB1 and ABCG2 transporters. *Sci. Rep.* 6:25694. 10.1038/srep25694 27157787 PMC4860574

[B46] Zhang Y. K. Zhang H. Zhang G. N. Wang Y. J. Kathawala R. J. Si R. (2015). Semi-synthetic ocotillol analogues as selective ABCB1-mediated drug resistance reversal agents. *Oncotarget* 6 24277–24290. 10.18632/oncotarget.4493 26296969 PMC4695185

[B47] Zhang Y. K. Zhang X. Y. Zhang G. N. Wang Y. J. Xu H. Zhang D. (2017). Selective reversal of BCRP-mediated MDR by VEGFR-2 inhibitor ZM323881. *Biochem. Pharmacol.* 132 29–37. 10.1016/j.bcp.2017.02.019 28242251 PMC7841404

